# Extracellular galectin-3 programs multidrug resistance through Na+/K+-ATPase and P-glycoprotein signaling

**DOI:** 10.18632/oncotarget.4285

**Published:** 2015-06-17

**Authors:** Yosuke Harazono, Dhong Hyo Kho, Vitaly Balan, Kosei Nakajima, Victor Hogan, Avraham Raz

**Affiliations:** ^1^ Departments of Oncology and Pathology, School of Medicine, Wayne State University, and Karmanos Cancer Institute, Detroit, MI 48201, USA; ^2^ Department of Maxillofacial Surgery, Tokyo Medical and Dental University, Bunkyo-ku, Tokyo 113-8510, Japan; ^3^ Everon Biosciences, Buffalo, NY 14203, USA

**Keywords:** galectin-3, Na^+^/K^+^-ATPase, multidrug resistance, P-glycoprotein, phosphorylation

## Abstract

Galectin-3 (Gal-3, LGALS3) is a pleotropic versatile, 29–35 kDa chimeric gene product, and involved in diverse physiological and pathological processes, including cell growth, homeostasis, apoptosis, pre-mRNA splicing, cell-cell and cell-matrix adhesion, cellular polarity, motility, adhesion, activation, differentiation, transformation, signaling, regulation of innate/adaptive immunity, and angiogenesis. In multiple diseases, it was found that the level of circulating Gal-3 is markedly elevated, suggesting that Gal-3-dependent function is mediated by specific interaction with yet an unknown ubiquitous cell-surface protein. Recently, we showed that Gal-3 attenuated drug-induced apoptosis, which is one of the mechanisms underlying multidrug resistance (MDR). Here, we document that MDR could be mediated by Gal-3 interaction with the house-keeping gene product e.g., Na^+^/K^+^-ATPase, and P-glycoprotein (P-gp). Gal-3 interacts with Na^+^/K^+^-ATPase and induces the phosphorylation of P-gp. We also find that Gal-3 binds P-gp and enhances its ATPase activity. Furthermore Gal-3 antagonist suppresses this interaction and results in a decrease of the phosphorylation and the ATPase activity of P-gp, leading to an increased sensitivity to doxorubicin-mediated cell death. Taken together, these findings may explain the reported roles of Gal-3 in diverse diseases and suggest that a combined therapy of inhibitors of Na^+^/K^+^-ATPase and Gal-3, and a disease specific drug(s) might be superior to a single therapeutic modality.

## INTRODUCTION

Galectins are a family of mammalian beta-galactoside binding proteins that share a highly conserved carbohydrate recognition domain (CRD) of which 15 galectin members have been identified to date [1, 2]. Galectin-3 (Gal-3) is the only member of the chimera-type galectin subgroup and contains a single CRD. There is both direct and circumstantial evidence that Gal-3 plays a significant role in cell growth, homeostasis, apoptosis, pre-mRNA splicing, adhesion, activation, differentiation, transformation, signaling, angiogenesis, inflammation, fibrosis, cancer progression, and metastasis [3–8].

It was reported that the level of circulating Gal-3 is significantly elevated in the serum of patients with various cancers [9–10, 11]. In addition, Gal-3 was found to be a potent pro-inflammatory protein and increased levels of serum Gal-3 were reported in immune and metabolic diseases, fibrosis, cirrhosis and heart diseases to name but few [3, 12, 13]. Gal-3 is lacking a leader sequence for secretion and is secreted into the extracellular *milieu via* non-classical pathways [7]. The interaction of Gal-3 with carbohydrate-conjugates of cell surface proteins and components of the extracellular matrix (ECM) such as MUC1, CD98, laminin and fibronectin, results in tumor cell migration, invasion and metastasis [14–16]. The binding of Gal-3 to alpha-v-beta-3 integrins and vascular endothelial growth factor (VEGF) receptor 2 on endothelial cells contributes to its pro-angiogenesis effect [16, 17]. Furthermore, extracellular soluble Gal-3 induces apoptosis of immune cells through the interaction with CD29 and CD7 [18]. Although multiple effects of circulating Gal-3 *via* the carbohydrate binding motif has been reported, it should be noted that Gal-3 directly interacts with proteins lacking carbohydrates, such as beta-catenin [19], Nup98 [20], Ras [21], U1 snRNP [22], Notch [23] and Bcl-2 family proteins [24, 25].

Multidrug resistance (MDR) phenotype is a major obstacle in successful chemotherapy. Cancer cells exhibit intrinsic or acquired MDR during tumor progression and/or drug therapy [26], and may develop a cross-drug-resistance to unexposed and structurally unrelated chemotherapeutic agents [27]. Several mechanisms underlying MDR were reported including decreased drug influx, increased drug efflux, altered cell cycle checkpoints, altered drug targets, increased drug metabolism and/or resistance to drug-induced apoptosis [26, 28]. Of these mechanisms, drug efflux is the most commonly encountered and mediated by ATP-binding cassette (ABC) transporters, such as the P-glycoprotein (P-gp/Mdr-1) [27]. Previously, we have reported that intracellular Gal-3 induced by drug treatment attenuates drug-induced apoptosis, a mechanism underlying MDR [25]. Others have reported that several secreted proteins like VEGF or SFRP contribute to the acquisition of MDR [29, 30], suggesting a possible role of secreted Gal-3 for MDR processes in cancer. Although several methods have been developed for targeting P-gp to avoid MDR, they only displayed limited success due to excessive systemic side effects [26].

In the present study, we embarked on a broad proteomic study to identify a cell surface binding-partner(s) of Gal-3, and found Na^+^/K^+^-ATPase. Furthermore we report extracellular Gal-3 enhances MDR phenotype through Na^+^/K^+^-ATPase and P-gp. The results reported here provide a new insight into the function of circulating Gal-3 in MDR processes.

## MATERIALS AND METHODS

### Cells

Human follicular thyroid carcinoma cells FTC-133 were obtained from the University of California Cell Culture Core Facility (San Francisco, CA). Human thyroid cells Nthy-ori 3–1 were purchased from Sigma-Aldrich (St. Louis, MO). Cervix adenocarcinoma epithelial cells HeLa, fibrosarcoma cells HT1080, breast cancer cells MDA-MB-231 and prostate cancer cells PC3 were purchased from American Type Culture Collection. These cell lines have been tested and authenticated by the supplier. All cells were cultured in Dulbecco's modified Eagle's medium (DMEM) supplemented with 10% fetal bovine serum (FBS), and maintained in a humidified chamber with 95% air and 5% CO_2_ at 37°C.

### Western blot assay

Cells were lysed in RIPA buffer (50 mM Tris-HCl pH 7.4, 1% NP-40, 0.5% Na-deoxycholate, 0.1% sodium dodecyl sulfate (SDS), 150 mM NaCl, 2 mM EDTA, 50 mM NaF and 0.2 mM Na_3_VO_4_) containing protease inhibitors (Roche Applied Science, Nutley, NJ). After BCA protein assay (Pierce Biotechnology, Rockford, IL), equal amounts of proteins were separated on 8% or 10% SDS-polyacrylamide gel electrophoresis (PAGE) gels and transferred to polyvinylidene fluoride membranes (Millipore, Bedford, MA). Membranes were blocked in 0.1% casein/Tris buffered saline (TBS) for 1 h, incubated with appropriate primary antibodies for overnight at 4°C, and then incubated with secondary antibodies conjugated with IRDye 800 (Rockland Immunochemicals, Gilbertsville, PA) or Alexa Fluor 680 (Invitrogen, Carlsbad, CA) for 1 h at room temperature. Membranes were washed three times with TBS including 0.1% Tween20 at 5-min intervals. Immunoblots were visualized, and the density of each band was quantitated using the Odyssey Infrared Imaging System and Odyssey application software (LI-COR Biosciences, Lincoln, NE). Each experiment was repeated at least, twice.

### Materials and antibodies

3, 3′-dithiobis sulfosuccinimidylpropionate (DTSSP) and beta-D-Lactose were purchased from Thermo Scientific (Pittsburgh, PA). Sucrose was purchased from MP Biomedicals (Solon, OH). Ouabain octahydrate, cis-diammineplatinum dichloride (CDDP), doxorubicin hydrochloride (DXR) and thiazolyl blue tetrazolium bromide (MTT) were purchased from Sigma-Aldrich. Pyrazolo pyrimidine (PP2), a Src kinase inhibitor, was purchased from Tocris Bioscience (Bristol, UK).

Customized polyclonal rabbit anti-Gal-3 antibody was created by Pierce Biotechnology; mouse anti-V5 and purified mouse IgG were purchased from Invitrogen; polyclonal goat anti-Na^+^/K^+^-ATPase alpha1 and monoclonal mouse anti-Mdr-1 were purchased from Santa Cruz Biotechnology (Santa Cruz, CA); polyclonal rabbit anti-Mdr was purchased from Oncogene Research Products (Cambridge, UK); mouse anti-beta-actin was purchased from Sigma-Aldrich; rabbit anti-Src and anti-p-Src were purchased from Cell Signaling Technology (Beverly, MA); purified rabbit IgG was purchased from ZYMED (San Francisco, CA); monoclonal mouse anti-phosphoserine was purchased from abcam (Cambridge, MA).

### MTT assay

Briefly, cells were seeded at well in 24-well plates. At the time of assay, 0.1 mg/ml MTT in basic medium was added to each well and incubated for 1 h. After removing MTT, dimethyl sulfoxide was added and mixed vigorously. Absorbance was measured at 485 nm.

### Preparation of recombinant Gal-3-V5 (rGal-3-V5)

The recombinant Gal-3 was constructed in the plasmid pET-22b (+) and expressed in E.coli BL21 (DE3) through inducement by IPTG. The protein was purified using lactosyl sepharose CL 4B column. Then, the protein was analyzed with Gelcode blue staining, concentrated in a buffer containing 20 mM Tris pH 7.9, 25% glycerol, 10 mM dithiothreitol (DTT), and 2 mM EDTA and stored at −80°C [31]. The concentration was measured by BCA protein assay.

### Proteomics analysis

Purified rGal-3-V5 was incubated with DTSSP cross linker in HeLa cells. Cells were lysed in buffer containing 1% 3-(3-cholamidopropyl) diethyl-ammonio-1-propanesulfonate (CHAPS) and protease inhibitors. Gal-3-V5-conjugated cell lysates were immunoprecipitated with anti-V5 antibody plus 15 alphal of protein G Sepharose (GE Healthcare, Pittsburgh, PA) overnight. After extensive washing, suspension in SDS sample buffer, and heat denaturing, the peptides were separated by 10% SDS-PAGE. Gel slices were transferred to The Wayne State University Proteomics Core Facility (The Wayne State University Proteomics Core Facility, job #1462). The peptides were separated by reverse phase chromatography (Acclaim PepMap100 C18 column), followed by ionization with the Nanospray Flex Ion Source, and introduced into a Q Exactive mass spectrometer (Thermo scientific). Data analysis was performed using Proteome Discoverer 1.4 (Thermo scientific) which incorporated the Mascot algorithm (Matrix Science, Boston, MA). The SwissProt_2013_04 database was used against human protein sequences and a reverse decoy protein database was run simultaneously for false discovery rate (FDR) determination. Secondary analysis was performed using Scaffold (Proteome Software, Portland, OR). Minimum protein identification probability was set as > = 99% with 2 unique peptides at > = 99% minimum peptide identification probability.

### Immunofluorescence

Cells were fixed with 4% paraformaldehyde in PBS for 15 min, permeabilized with 1% Saponin for 10 min, blocked with 1% BSA in PBS for 30 min and incubated with indicated antibodies for overnight, then incubated with tetramethylrhodamine isothiocyanate (TRITC)-conjugated antibody and fluorescein isothiocyanate (FITC)-conjugated antibody (Sigma-Aldrich) for 1 h in the dark. Nuclei were stained with 1 microgram/ml of 4′, 6-diamidino-2-phenylindole (DAPI) for 3 min. Pictures were taken using a Zeiss Confocal Laser Microscope LSM 510 META NLO (The Wayne State University Microscopy and Imaging Core Facility).

### Co-immunoprecipitation assay

Cells were lysed in previously described buffer containing 1% CHAPS and protease inhibitors. After BCA protein assay, cell lysates containing equal amount of proteins were incubated with appropriate antibodies overnight and 15 microliters of protein G Sepharose (GE Healthcare) for 1 h at 4°C. The beads were washed three times and boiled in 2x sample buffer. Supernatant was subjected to SDS-PAGE and immunoblotted for appropriate antibodies. Each experiment was repeated at least, twice.

### siRNA transfection

siRNA against Na^+^/K^+^-ATPase alpha(sc-43956) and Mdr-1 (sc-29395), and control siRNA (sc-37007) (Santa Cruz Biotechnology) were transfected into each cells using Lipofectamine RNAiMax reagent (Invitrogen) according to the manufacturer's instruction. Cell viability after transfection was determined by the MIT assay, see above, and both the control and the experimental assays were found to yield > 95% cell viability.

### Preparation of GCS-100/modified citrus pectin (MCP)

Citrus Pectin (CP) was purchased from Sigma Chemicals. Temperature modification of CP was performed as follows: CP solution (1.3%) was autoclaved for 1 h; cooled to room temperature, centrifuged at 10, 000 *g* for 10 min. collected supernatant was precipitated with 2 volumes of absolute ethanol and frozen at −20°C for 2 h. After centrifuging at 10, 000 *g* for 10 min again, the supernatant was discarded and pellet was saved. The pellet was suspended in acetone, filtered, and dried on Whatman filters. MCP was dissolved in de-ionized distilled water.

### P-gp ATPase activity assay

The P-gp-Glo™ Assay Systems (Promega, Madison, WI) was used to perform P-gp ATPase activity assay. A P-gp ATPase assay detects the effect of compounds on recombinant human P-gp in the cell membrane fraction. Briefly, ATP was first incubated with P-gp membranes and MgATP, then the P-gp ATPase reaction was stopped, and the remaining ATP was detected as a luciferase-generated luminescent signal. Basal P-gp ATPase activities were investigated by detecting activity in the presence or absence of sodium orthovanadate. The P-gp-GloTM Assay Systems (Promega, Madison, WI) was used, as described [32] to perform not total ATPase but P-gp ATPase activity assay with recombinant P-gp. This commercial assay kit includes Verapamil as a positive control for P-gp activity. The converted drug-stimulated ATPase activity (nmol ATP consumed/mg P-gp/min) was determined by comparing the luminescence of the samples.

### Statistical analysis

Statistical analysis was done using two-tailed Student *t*-test. *P* < 0.05 was regarded as significant. A single asterisk indicates a *P* < 0.05, whereas a double asterisk indicates a *P* < 0.01.

## RESULTS

### Doxorubicin (DXR) induces Gal-3 secretion and extracellular Gal-3 interacts with Na^+^/K^+^-ATPase

**Figure 1:**
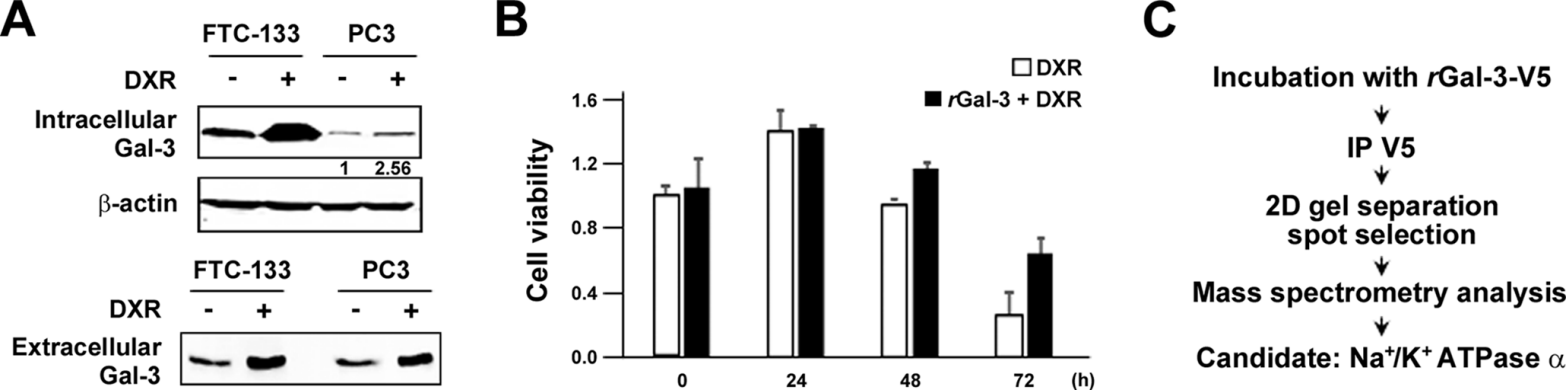
**A. FTC-133 and PC3 cells were seeded, and 24 h later, media were changed to media lacking FBS.** At the same time, cells were either left untreated or treated with 0.5 micromolar DXR for 6 h. Western blot analysis shows intracellular and extracellular Gal-3 expression in total cell lysates (*Upper panel*) and conditioned media (*Lower panel*), respectively. The protein in conditioned media was concentrated with 3K centrifugal filters (Millipore). beta-actin was used as the loading control for total cell lysates. Numbers represent the relative intensity of intracellular Gal-3 to beta-actin in PC3. The value of untreated PC3 cells was set as 1. **B.** PC3 cells were seeded, and 24 h later, media were changed to media lacking FBS (0 h). At the same time, cells were treated with 0.1 micromolar DXR in the presence or absence of 0.5 micromolar purified recombinant Gal-3-V5 (rGal-3-V5). Cell viability was determined by MTT at the indicated time. The relative value of untreated cells at 0 h was set as 1. *Columns* represent the mean of two independent experiments; *bars*, SE. **C.** rGal-3-V5 was incubated with DTSSP cross linker in cells. Gal-3-V5-conjugated cell lysates were immunoprecipitated with V5 antibody and then subjected to SDS-PAGE. 20 gel slices harboring Gal-3-V5-interacting candidates were analyzed with mass spectrometry as in Material and Methods. Na^+^/K^+^-ATPase alpha1 was selected as a candidate protein.

**Figure 2:**
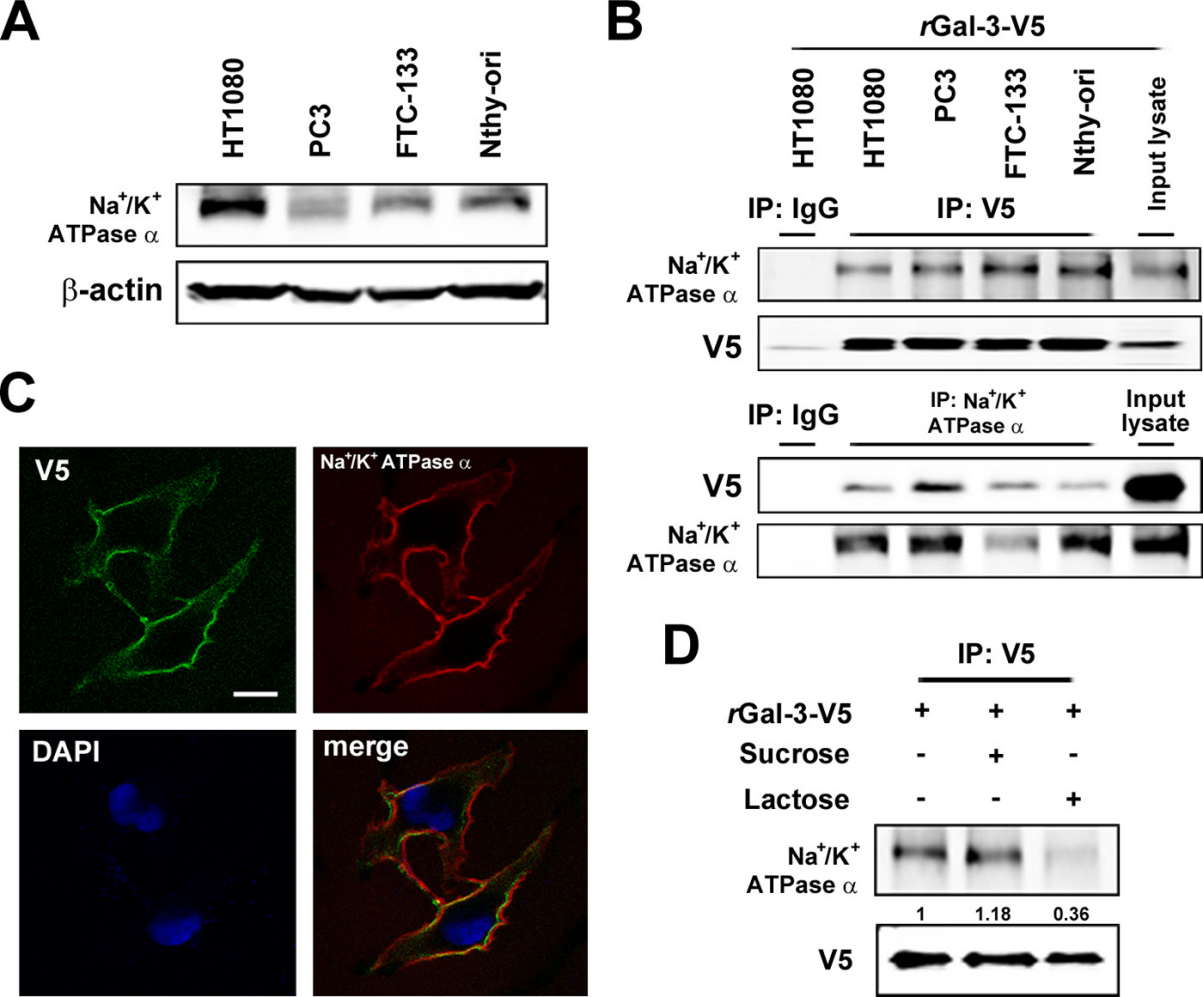
**A. Western blot analysis shows Na^+^/K^+^-ATPase alpha1 protein expression in the indicated cell lysates.** beta-actin was used as the loading control. **B.** Co-immunoprecipitation assay. Cells were incubated with 0.5 micromolar rGal-3-V5 for 3 h. The indicated cell lysates were incubated with 0.5 micromolar DTSSP for 30 min and immunoprecipitated with IgG, V5 or Na^+^/K^+^-ATPase alpha1 antibody. Immunoprecipitates were analyzed by immunoblotting with indicated antibodies. Input lysates treated with rGal-3-V5 was used as control. **C.** Co-localization of Gal-3-V5 and Na^+^/K^+^-ATPase alpha1 in Nthy ori 3–1 cells treated with 1 micromolar rGal-3-V5 for 3 h was observed by confocal microscopy. Cells were immunofluorescent labelled with anti-V5 (green), anti-Na^+^/K^+^-ATPase alpha1 (red) antibodies and DAPI (nuclear stain, blue). Scale bar represents 20 micrometers. **D.** HT1080 cells were either untreated or treated with 200 mM of sucrose (used as sugar control) or lactose, 1 h later, treated with 1 micromolar rGal-3-V5, depicting the co-localization of Na/K ATPase and Gal-3 on the cell surface. Cell lysates were incubated with 0.5 micromolar DTSSP for 30 min and immunoprecipitated with V5 antibody. Immunoprecipitates were analyzed by immunoblotting with Na^+^/K^+^-ATPase alpha1 or V5 antibody. Numbers represent the relative intensity of Na^+^/K^+^-ATPase alpha1 normalized to V5. The value of cells treated with rGal-3-V5 alone was set as 1.

Gal-3 overexpression in cancer cells leads to its higher secretion and concentration in the circulation of patients [7, 8, 33]. Our previous report indicated that patients with metastases arising from prostate cancer have a higher serum galectin-3 concentration [12]. In addition, we recently showed that the anti-apoptotic function of intracellular Gal-3 in which its expression increased with cis-diammineplatinum dichloride (CDDP) and DXR treatment [25]. Thus, we questioned whether extracellular Gal-3 also contributes to the protection of cancer cells against anti-cancer drug. Initially, we examined its expression level after DXR treatment. As expected, we detected an increase of expression and secretion of Gal-3 in cells treated with DXR (Figure 1A). To address the role of extracellular Gal-3 on cell survival, we investigated cell viability with DXR treatment in the presence or absence of 0.5 micromolar exogenous recombinant Gal-3-V5 (rGal-3-V5) protein. As shown in Figure 1B, cells treated with rGal-3-V5 showed increased viability against DXR treatment. Next, to identify a cell surface molecule(s) interacting with Gal-3, we performed a broad proteomic study using mass spectrum following the co-immunoprecipitation with purified rGal-3-V5. As a result, 350 proteins were nominated from the analysis and Na^+^/K^+^-ATPase alpha1 subunit (NCB Accession # P05023) was found to be the most specific and therefore selected as a candidate protein for further studies to identify if it is responsible for exogenous Gal-3-induced drug resistance (Figure 1C). Of note, others have reported that alterations in the homeostasis of Na^+^ and K^+^ ions cause change of cell volume and lead to apoptosis [28]. Furthermore, in drug-induced apoptotic processes, Na^+^/K^+^-ATPase may act as an anti-apoptotic transporter [34]. Thus, we examined the expression pattern of Na^+^/K^+^-ATPase in several cancer cells (Figure 2A). To further confirm Gal-3-Na^+^/K^+^-ATPase interaction, we performed co-immunoprecipitation assays using total cell lysates from cells treated with rGal-3-V5. As predicted, the endogenous Na^+^/K^+^-ATPase was co-precipitated with Gal-3-V5 regardless of cancer cell type, and the reciprocal experiments revealed that Gal-3-V5 was co-immunoprecipitated with Na^+^/K^+^-ATPase (Figure 2B). In addition, confocal immunofluorescent microscopic visualization depicted a co-localization of Gal-3-V5 and Na^+^/K^+^-ATPase on the cell surface (Figure 2C). To further delineate whether the interaction is dependent on the sugar-binding properties of Gal-3, we added lactose, a Gal-3 sugar antagonist [18], to cells and found that the interaction of Gal-3-V5 with Na^+^/K^+^-ATPase in cells treated with lactose was significantly reduced as compared with control (Figure 2D). These data indicate that Gal-3-Na^+^/K^+^-ATPase recognition and binding is through the CRD of Gal-3.

### Gal-3 activates Src kinase through the interaction with Na^+^/K^+^-ATPase

**Figure 3:**
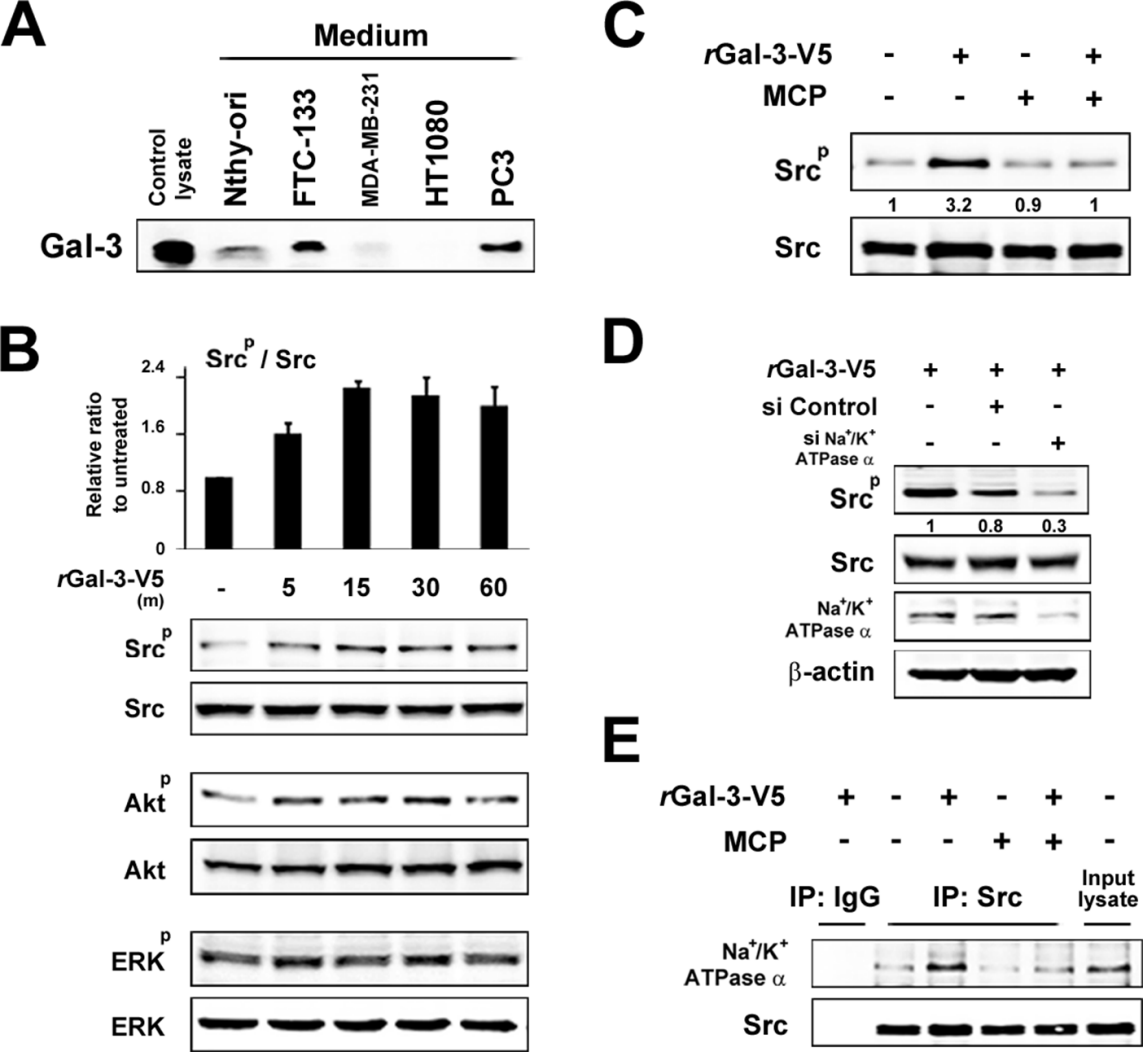
**A. The indicated cell lines were starved for 24 h in media lacking FBS.** The protein in conditioned media was concentrated with 3K centrifugal filters. After BCA assay, equal amounts of proteins were separated using SDS-PAGE. Western blot analysis shows Gal-3 expression in conditioned media with polyclonal anti-Gal-3 antibody. **B.** HT1080 cells were starved for 24 h in media lacking FBS and then treated with 0.5 micromolar rGal-3-V5. Kinetic analyses were done at the times indicated. Analyses of p-Src were normalized to Src (*Upper panel* ). The relative value of untreated cells was set as 1. *Columns* represent the mean of two independent experiments; *bars*, SE. Western blot analyses show the representative data of p-Src, p-Akt and p-ERK (*Lower panel* ). **C.** HT1080 cells were either left untreated or treated with 1% of GCS-100/MCP, 30 min later, treated with 0.5 micromolar rGal-3-V5 for 15 min. Western blot analyses were performed with p-Src or Src antibody. Numbers represent the relative intensity of p-Src normalized to Src. The value of untreated cells was set as 1. **D.** HT1080 cells were transfected with 10 nM of si-Control or si-Na^+^/K^+^-ATPase alpha1 for 24 h, and then starved in media lacking FBS. At the same time, they were either untreated or treated with 0.5 micromolar rGal-3-V5 for 15 min. Western blot analyses were performed with p-Src, Src or Na^+^/K^+^-ATPase alpha1 antibody beta-actin was used as the loading control. Numbers represent the relative intensity of p-Src normalized to Src. The value of cells treated with rGal-3-V5 alone was set as 1. **E.** HT1080 cells were treated as in C. Cell lysates were immunoprecipitated with IgG or Src antibody. Immunoprecipitates were analyzed by immunoblotting with Na^+^/K^+^-ATPase alpha1 or Src antibody. Input lysate from HT1080 was used as control.

**Figure 4:**
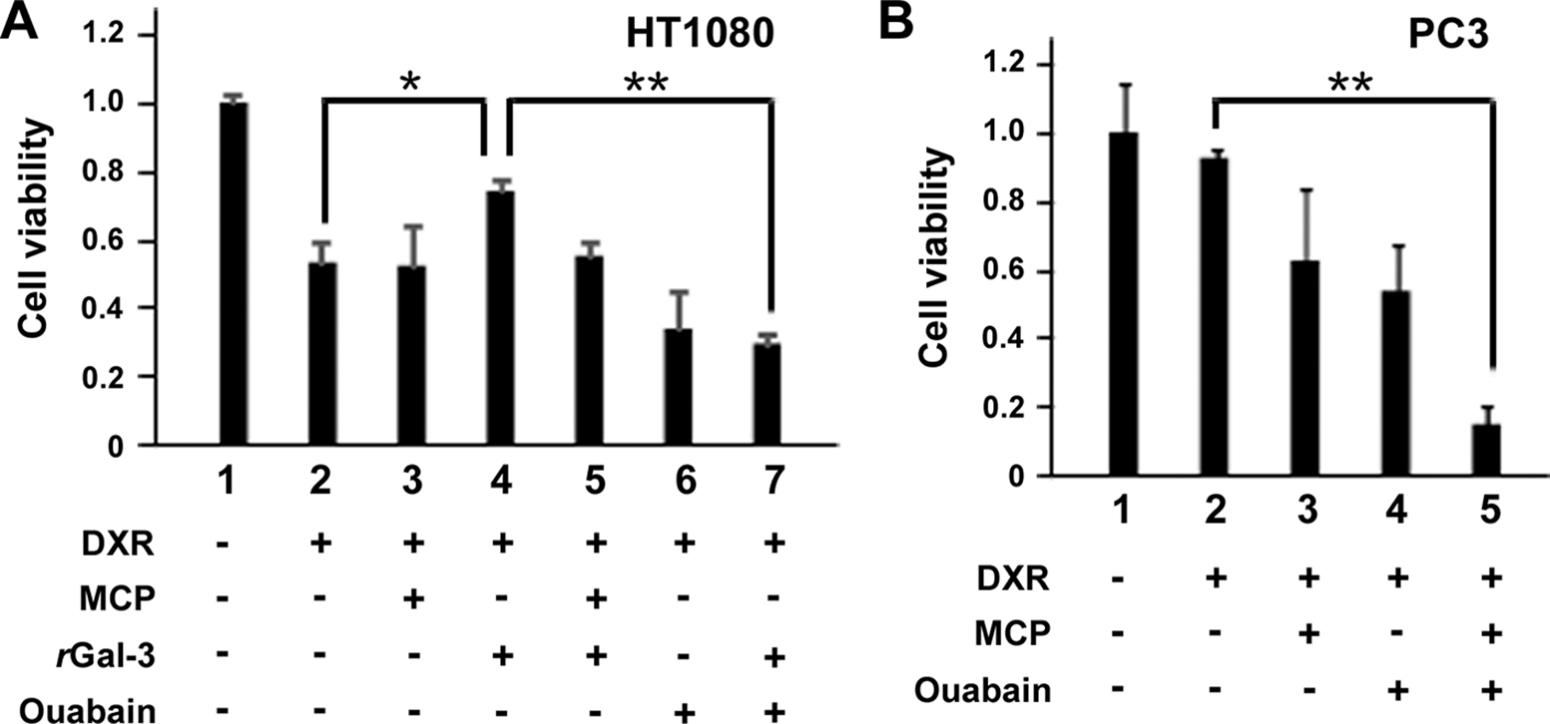
**A and B. HT1080 and PC3 cells were seeded, and 24 h later, media were changed to media lacking FBS.** At the same time, cells were either left untreated or treated with 0.1 micromolar DXR, 0.5 micromolar rGal-3-V5, 1% of GCS-100/MCP, 100 nM of ouabain or a combination. Cell viability was determined by MTT, 24 h later. The relative value of untreated cells was set as 1. **P < 0.01, *P < 0.05. *Columns* represent the mean of three independent experiments; *bars*, SE.

Next we explored the molecular outcomes of the interaction of Gal-3 with Na^+^/K^+^-ATPase. Apart from ion homeostasis, Na^+^/K^+^-ATPase was reported to interact with Src, membrane-associated non-receptor tyrosine kinases, and form a functional signal complex [34]. Na^+^/K^+^-ATPase relays the extracellular signal to intracellular compartments *via* cascades of EGFR-Src-ERK [36]. In this mechanism, as Src kinase is trapped in Na^+^/K^+^-ATPase and is able to be activated by the ligand of Na^+^/K^+^-ATPase [34, 37], we examined whether the interaction of Gal-3 with Na^+^/K^+^-ATPase was also associated with the activation of Src kinase. We have tested human fibrosarcoma cells HT1080 since they secret very low level of endogenous Gal-3 (Figure 3A). The incubation with rGal-3-V5 significantly increased the phosphorylation of Src and Akt, with no significant change of ERK phosphorylation (Figure 3B). This phosphorylation of Src was suppressed by a clinically tested Gal-3 antagonist *e.g*., modified citrus pectin (MCP) [25, 38, 39] (Figure 3C). To address whether Gal-3-induced Src phosphorylation was mediated by Na^+^/K^+^-ATPase, we treated the cells with siRNA of Na^+^/K^+^-ATPase and found that Gal-3-induced Src phosphorylation was suppressed in Na^+^/K^+^-ATPase knockdown cells (Figure 3D). Finally, we questioned whether Gal-3 affects the interaction of Na^+^/K^+^-ATPase and Src. It was found that Na^+^/K^+^-ATPase was co-precipitated with Src after incubation with rGal-3-V5 and MCP suppressed this interaction (Figure 3E). These data indicate that Gal-3 activates Src and induces Src-Na^+^/K^+^-ATPase interaction. To address whether Gal-3-induced cell survival is mediated by Na^+^/K^+^-ATPase, we have tested the effects of both MCP and ouabain, an inhibitor of Na^+^/K^+^-ATPase, on DXR-treated cells. As shown in Figure 4A, exogenous Gal-3 partially recovered the cell viability of DXR-treated cells and this resistance was reduced by ouabain, suggesting that Na^+^/K^+^-ATPase is a downstream effector of Gal-3, as concluded from the fact that MCP and/or ouabain affect DXR-mediated cell death (Figure 4B).

### Gal-3 induces phosphorylation of P-gp via Na^+^/K^+^-ATPase

**Figure 5:**
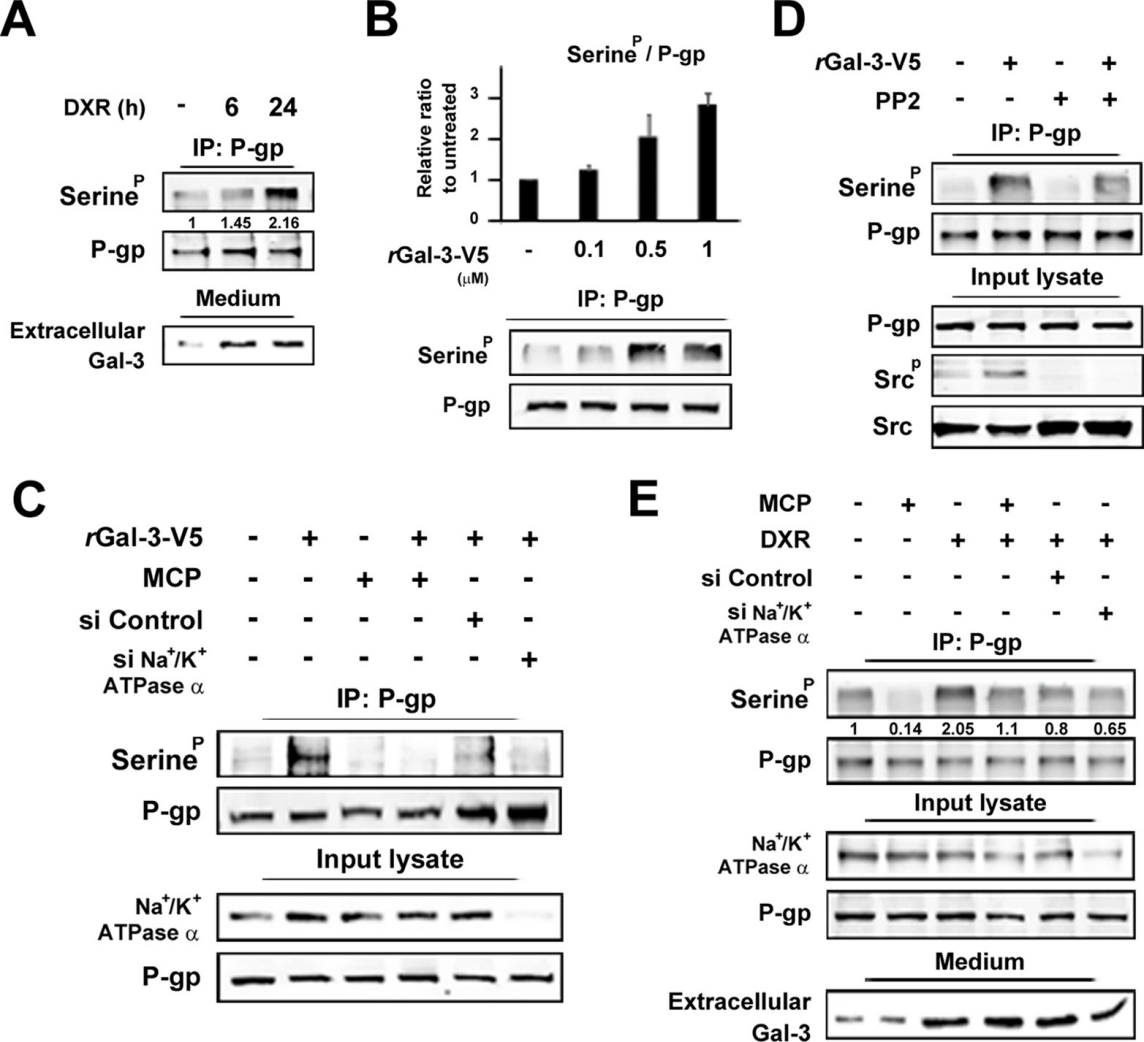
**A. PC3 cells were starved in media lacking FBS.** At the same time, cells were either left untreated or treated with 0.5 micromolar DXR for indicated times. Cell lysates were immunoprecipitated with P-gp antibody. Immunoprecipitates were analyzed by immunoblotting with p-Serine or P-gp antibody. The protein in conditioned media was concentrated with 3K centrifugal filters. After BCA assay, equal amounts of proteins were separated using SDS-PAGE. Numbers represent the relative intensity of p-Serine normalized to P-gp. The value of untreated cells was set as 1. **B.** HT1080 cells were starved for 24 h in media lacking FBS and then treated with of rGal-3-V5 for the indicated concentrations for 24 h. Cell lysates were immunoprecipitated and analyzed as in A. Analyses of p-Serine were normalized to P-gp (*Upper panel *). The relative value of untreated cells was set as 1. *Columns* represent the mean of two independent experiments; *bars*, SE. Western blot analyses show the representative data (*Lower panel *). **C.** HT1080 cells were transfected with 10 nM of si-Control or si-Na^+^/K^+^-ATPase alpha1 for 24 h, and then starved in media lacking FBS. At the same time, cells were either left untreated or treated with 0.5 micromolar rGal-3-V5 in the presence or absence of 1% of GCS-100/MCP for 24 h. Cell lysates were immunoprecipitated and analyzed as in A. Input lysate from HT1080 was used as control. **D.** HT1080 cells were starved in media lacking FBS. At the same time, cells were treated with 0.5 micromolar rGal-3-V5 in the presence or absence of 10 micromolar PP2 for 24 h. Cell lysates were immunoprecipitated and analyzed as in A. Input lysate from HT1080 was used as control. **E.** PC3 cells were transfected with 10 nM of si-Control or si-Na^+^/K^+^-ATPase alpha1 for 24 h, and then starved in media lacking FBS. At the same time, cells were either left untreated or treated with 0.5 micromolar DXR in the presence or absence of 1% of GCS-100/MCP for 24 h. Cell lysates and the protein in conditioned media were analyzed as in A. Input lysate from PC3 was used as control. Numbers represent the relative intensity of p-Serine as in A.

Since P-gp is a molecular pump protecting cells from toxic molecules [26], we examined P-gp expression in PC3 cells treated with DXR. Although we did not observe any change in the level of P-gp expression, its phosphorylation was induced in response to DXR treatment, suggesting that Gal-3 affects P-gp phosphorylation (Figure 5A). Next we determined the phosphorylation status of P-gp with the incubation of rGal-3-V5 at the indicated concentrations and exogenous Gal-3 induced P-gp phosphorylation in a concentration-dependent manner (Figure 5B). Furthermore, we examined the possible suppression effect on P-gp phosphorylation by Gal-3 antagonist or siRNA against Na^+^/K^+^-ATPase and both markedly suppressed P-gp phosphorylation induced by Gal-3 (Figure 5C). Taking into account that Gal-3 activates Src kinase through Na^+^/K^+^-ATPase (Figure 3), we utilized PP2, a Src kinase inhibitor to determine the effect of Src kinase on P-gp phosphorylation (Figure 5D). Indeed, PP2 suppressed the induction of P-gp phosphorylation with rGal-3-V5 treatment, indicating that Gal-3 induces phosphorylation of P-gp *via* Na^+^/K^+^-ATPase and Src kinase. Moreover, in PC3 cells treated with DXR, the P-gp phosphorylation level was increased. This induction was suppressed by MCP or inhibition of Na^+^/K^+^-ATPase, suggesting the effect of endogenous secreted Gal-3 (Figure 5E).

### Gal-3 confers cell resistance to DXR through activation of P-gp

**Figure 6:**
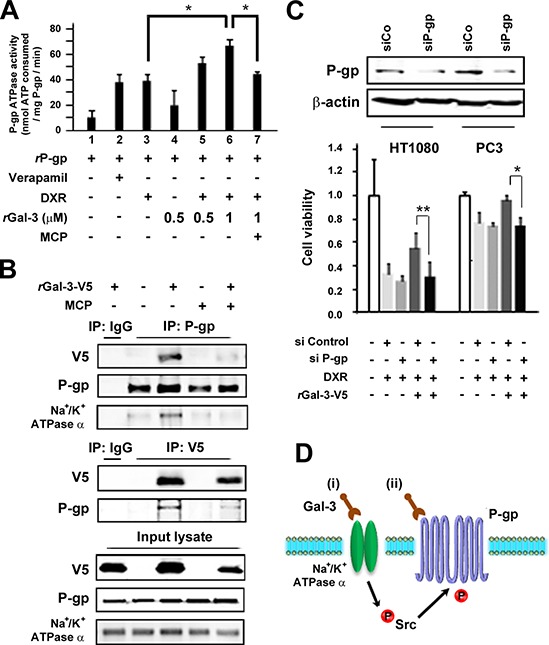
**A. Effect of Gal-3 on the P-gp ATPase activity in human P-gp-expressing membranes.** Membranes were treated with Verapamil (200 micromolar), DXR (5 micromolar), recombinant Gal-3 (0.5 micromolar or 1 micromolar) or GCS-100/MCP (1%). The differences in sample luminescence were used to calculate ATPase activity, which was expressed as nanomoles of ATP consumed in reaction. *Columns* represent the mean of three independent experiments; *bars*, SE. **B.** Co-immunoprecipitation assay. HT1080 cells were seeded and media were changed to media lacking FBS. At the same time, cells were either left untreated or treated with 0.5 micromolar rGal-3-V5 in the presence or absence of 1% of GCS-100/MCP for 24 h. Cell lysates were immunoprecipitated with IgG, P-gp or V5 antibody. Immunoprecipitates were analyzed by immunoblotting with V5, P-gp or Na^+^/K^+^-ATPase alpha1 antibody. Input lysate from HT1080 was used as control. **C.** HT1080 and PC3 cells were transfected with 10 nM of si-Control or si-P-gp for 24 h, and then starved in media lacking FBS. At the same time, cells were either left untreated or treated with 0.1 micromolar DXR in the presence or absence of 0.5 micromolar rGal-3-V5 for 48 h. Western blot analysis shows P-gp expression in control or P-gp knockdown cells (*Upper panel *). Cell viability was determined by MTT (*Lower panel *). The relative value of untreated cells was set as 1. **P < 0.01, *P < 0.05. *Columns* represent the mean of three independent experiments; *bars*, SE. **D.** Schematic representation for the regulation of P-gp by extracellular Gal-3. (i) Gal-3 activates Src kinase through the interaction with Na^+^/K^+^-ATPase, subsequently leading to the induction of phosphorylation of P-gp. (ii) Gal-3 binds P-gp and induces the ATPase activity of P-gp. As a result, Gal-3 contributes to the acquisition of MDR through Na^+^/K^+^-ATPase and P-gp.

To determine whether Gal-3 affects P-gp activity, drug-stimulated P-gp ATPase activity assay was performed. The function of P-gp is connected to ATP hydrolysis by the ATPase, and its activity is influenced by P-gp substrates or modulators. As shown in Figure 6A, the incubation of Gal-3 with P-gp enhanced the P-gp ATPase activity stimulated by DXR (lane 3 and 6), while addition of MCP suppressed the enhancement of ATPase activity (lane 7), indicating that Gal-3 directly affects the ATPase activity of P-gp. Thus, we expected that Gal-3 bound P-gp and performed co-immunoprecipitation assay. As shown in Figure 6B, Gal-3-V5 was co-precipitated with P-gp (*Upper Panel*), the reciprocal experiments revealed that P-gp was co-immunoprecipitated with Gal-3-V5 (*Middle Panel*) and this interaction was depend on CRD of Gal-3. Intriguingly, we also noted a co-precipitation of Na^+^/K^+^-ATPase with P-gp (*Upper Panel*). Finally, we examined cell viability with rGal-3-V5 and DXR treatment in P-gp knockdown cells (Figure 6C). The exogenous Gal-3 partially recovered the cell viability in DXR-treated cells and the resistance was suppressed in P-gp knockdown cells. These data show for the first time that Gal-3 is involved in cell resistance to cytotoxic drug through activation of P-gp.

## DISCUSSION

Here, we report that extracellular Gal-3 contributes to the acquisition of MDR through interaction with Na^+^/K^+^-ATPase and P-gp in cancer cells. Circulating Gal-3 serves as a biomarker for monitoring recurrence/metastasis in several cancers [9–11] and the role of Gal-3 on MDR was revealed in this cell-based study. Therefore, the consideration of targeting extracellular Gal-3 may take advantage of an improved therapeutic modality due to inactivation of P-gp and circumvention of MDR.

Na^+^/K^+^-ATPase is composed of three subunits, regulates the translocation of Na^+^ and K^+^ across the cell membrane and acts as a signal transducer [40]. The expression of major isoform of Na^+^/K^+^-ATPase, subunit alpha1 is ubiquitously detected in various tissues of all higher eukaryotes [41]. Inhibition of Na^+^/K^+^-ATPase has been widely used for the treatment of heart failure. Also, it was a reduction of cancer incidence in cardiac patients taking digoxin, was observed together with experimental and epidemiological studies have consolidated the anti-cancer potential of Na+/K+ ATPase inhibitors (for review see 42). More importantly, a new series of pharmacologically optimized Na^+^/K^+^-ATPase inhibitors have recently shown strong anti-cancer activities and reached early clinical trials [42–44]. Further it was documented and proposed that Na (+)/K (+)-ATPase is a valuable target in anticancer therapy and its inhibitors and ligands could be designed as potential anticancer agents. Of note, a recent review [45] has noted that clinical trials with Na(+)/K(+)-ATPase as a single agent or in combination with other anticancer were safe but showed limited efficacy in cancer patients Thus, it was recommended that well-designed randomized clinical trials s are needed to confirm the efficacy and safety of cardiac glycosides for the treatment of cancer. We found that Gal-3 bound a novel binding partner, Na^+^/K^+^-ATPase. The finding that Na^+^/K^+^-ATPase interacts with circulating Gal-3 is further supported by earlier reports, showing that Na^+^/K^+^-ATPase reacts with beta-galactoside soybean agglutinin, peanut agglutinin [46] and cholera toxin (CT) [47]. Of note, there are several functional similarities between Gal-3 and Na^+^/K^+^-ATPase, including apoptosis [24, 45], inflammation [6, 49], ERK signaling [31, 50], membrane recycling [51, 52] and anoikis [53, 54] and both are associated with the scavenger receptor CD36 [52, 55]. Since serum Gal-3 is an emerging biomarker for cancers and other inflammation-related diseases [6–8, 32]. Of note, recently published report that has concluded that “the formation of Na/K-ATPase-Src complex might enable a dynamic on/off mechanism of Src regulation, and that the α1 Na/K-ATPase may be a key player in dynamic regulation of cellular Src activity [55]. Further, it was shown that cells contain a pool of Src-interacting Na/K-ATPase that not only regulates Src activity but also serves as a receptor for ouabain to activate protein kinases (56 leading ; showing Binding of Src to the formation of Na+/K+-ATPase a functional signaling complex all confirming our assertion. And requiring that future studies of Gal-3-Na^+^/K^+^-ATPase biology would include Gal-3/Na^+^/K^+^-ATPase/Src axis consideration as it may provide new insight to understand the pathophysiology of multiple Gal-3-overexpressing diseases such as immune diseases, metabolic diseases and fibrosis as well as cancer. It should also be noted that the existence of Na/K ATPase ‘signalosome’ was implicated to be interacts with major components of gliomagenesis: EGFR, caveolin-1, PI3K, Src, and Ras. On ouabain binding, the NaK α1 subunit in caveolae associates with several proteins, such as caveolin-1 [57]. In addition a Gal-3-Src interaction was reported as well [58, 59]. The above and the present findings thus, give credence to the further need to determine the functional relationship between Gal-3 and Na^+^/K^+^-ATPase and their inhibitors in the clinical setting.

P-gp binds hydrophobic molecules in the cell membrane, and then undergoes a conformational alteration. The new conformation opens toward the outside of the cell, and the molecule is ejected utilizing ATP as the driving force [26]. P-gp is known to be regulated by post-translational modification as well as transcription. It has serine phosphorylation residues in the linker region between the two homologous halves, mediated by protein kinase A (PKA) and protein kinase C (PKC) [60]. Na/k ATPase was shown to be associated with the rug resistance phenotype [61], and a down-regulation of Na+/K+-ATPase in multidrug-resistant, P-glycoprotein-overexpressing cells was observed [62]. The downstream signaling pathways were deregulated in the Na+/K+-ATPase ‘signalosome’ and to play a role be in the multidrug-resistant phenotype [63]. It was also shown that anti-Na/K ATPase stimulates the expression of the MDR-1 (multidrug resistance) gene and the synthesis of an active P-glycoprotein (P-gp) in some human epithelial cells, these data, which emphasize the complex mechanism of action of ouabain, suggest that changes in intracellular ionic activities modulate CFTR/MDR-1 gene expressions [54]. It was also suggested that Glioblastoma patients who do not respond to chemotherapy and whose tumors over-express NaK alpha1 subunits could benefit from a treatment using ligands with marked binding affinity for the NaK alpha1 subunit [57].

P-gp phosphorylation has been reported to be essential for its function [64], while controversy exists as other reports have suggested its phosphorylation has no effect on the function of P-gp [65]. Although we identified a tyrosine kinase, Src-mediated serine phosphorylation of P-gp in Gal-3-treated cells, further studies including the identification of specific phosphorylation sites and the involved serine kinases, are needed to establish the functional relationship between activity and phosphorylation of P-gp.

Based on the above data, we propose the following model for the mechanisms by which extracellular Gal-3 involves in MDR depicted in Figure 6D: (i) Gal-3 activates Src kinase through the interaction with Na^+^/K^+^-ATPase, subsequently leading to the induction of phosphorylation of P-gp; (ii) Gal-3 also binds P-gp and induces the ATPase activity of P-gp. However, the question includes whether these mechanisms are independent or Gal-3/Na^+^/K^+^-ATPase/P-gp cooperate in acquiring MDR phenotype. Recently, targeting Na^+^/K^+^-ATPase was reported to allow circumvention of various chemo resistance pathways [65], all of which point to a correlation between Na^+^/K^+^-ATPase and P-gp in the expression of MDR phenotype.

Most clinical trials targeting P-gp fail due to toxic side effects and pharmacokinetic interactions that limited drug clearance and metabolism of chemotherapy [26]. Although it is generally accepted that cancer cells can convert to become multidrug resistant, normal human tissues remain sensitive to the toxic effects of chemotherapy, suggesting that multidrug-resistance gene products might be useful for protection of normal tissues against the cytotoxic side effects of anti-cancer drug [27]. Accordingly, targeting selectively circulating Gal-3 in cancer using its antagonist may provide a new therapeutic paradigm for circumvention of MDR without side effects obtained from direct inhibition of P-gp. A combined therapy of inhibitors of Na+/K+-ATPase and Gal-3, and a disease specific drug(s) may be superior to a single therapeutic modality.
